# Enhancing the efficacy of cytotoxic agents for cancer therapy using photochemical internalisation

**DOI:** 10.1002/ijc.29510

**Published:** 2015-03-23

**Authors:** Alejandra Martinez de Pinillos Bayona, Caroline M. Moore, Marilena Loizidou, Alexander J. MacRobert, Josephine H. Woodhams

**Affiliations:** ^1^ UCL Division of Surgery and Interventional SciencesUniversity College LondonLondonUnited Kingdom

**Keywords:** photochemical internalisation, photodynamic therapy, drug delivery system, drug resistance, chemotherapy

## Abstract

Photochemical internalisation (PCI) is a technique for improving cellular delivery of certain bioactive agents which are prone to sequestration within endolysosomes. There is a wide range of agents suitable for PCI‐based delivery including toxins, oligonucleotides, genes and immunoconjugates which demonstrates the versatility of this technique. The basic mechanism of PCI involves triggering release of the agent from endolysosomes within the target cells using a photosensitiser which is selectively retained with the endolysosomal membranes. Excitation of the photosensitiser by visible light leads to disruption of the membranes *via* photooxidative damage thereby releasing the agent into the cytosol. This treatment enables the drugs to reach their intended subcellular target more efficiently and improves their efficacy. In this review we summarise the applications of this technique with the main emphasis placed on cancer chemotherapy.

**Abbreviations**
DDS: drug delivery system
EGFR: endothelial growth factor receptor
EGP‐2: endothelial glycoprotein‐2
HER‐2: human epidermal growth factor receptor 2
HUVECs: human vascular endothelial cells
MDR: multidrug resistance
NP: nanoparticle
PCI: photochemical internalisation
PDT: photodynamic therapy
PS: photosensitiser
RIP: ribosome inactivating protein
ROS: reactive oxygen species

Chemotherapy is routine in the treatment of a range of cancers, however limitations include systemic toxicity and multidrug resistance (MDR),^1^, ^2^ and in some cases inadequate cellular delivery.^3^, ^4^ These drawbacks have stimulated considerable effort into devising new drug delivery systems (DDS) for cancer chemotherapy agents including “active” DDS that rely on an externally applied energy input such as a local electric field (irreversible electroporation) or ultrasound.^5^, ^6^, ^7^ Photochemical Internalisation was originally conceived by Berg *et al*. and is also an “active” DDS, since drug delivery is triggered by the application of light.^8^, ^9^ A key advantage of such active DDS like PCI is that the drug delivery process can be triggered at the optimum time following drug administration.

### Mechanism of PCI

The basic mechanism of PCI involves triggering drug release from endolysosomes within the target cells using a photosensitiser (PS) which is selectively retained with the endolysosomal membranes. Excitation of the PS using visible light in the presence of molecular oxygen leads to disruption of organelle membranes *via* photooxidative damage thereby releasing the drug confined within the organelles (Fig. 1 *a*). This treatment enables the drugs to reach their intended subcellular target more efficiently which enhances their efficacy. This release mechanism also serves to counteract enzyme‐induced degradation of some drugs within lysosomes. Because photosensitisers used for PCI are fluorescent, the photochemically induced disruption to the endolysosomes can be studied using fluorescence imaging.^8^, ^9^ Release of fluorescent nanoparticles (NPs) such as quantum dots initially confined to the endolysosomal central aqueous compartment concomitant with redistribution of the PCI photosensitiser has been observed *in vitro*.^11^ Woodhams *et al*. have also demonstrated *in vivo* light‐induced redistribution of a cytotoxic agent, gelonin, in treated rat liver using immunohistochemistry.^12^

**Figure 1:**
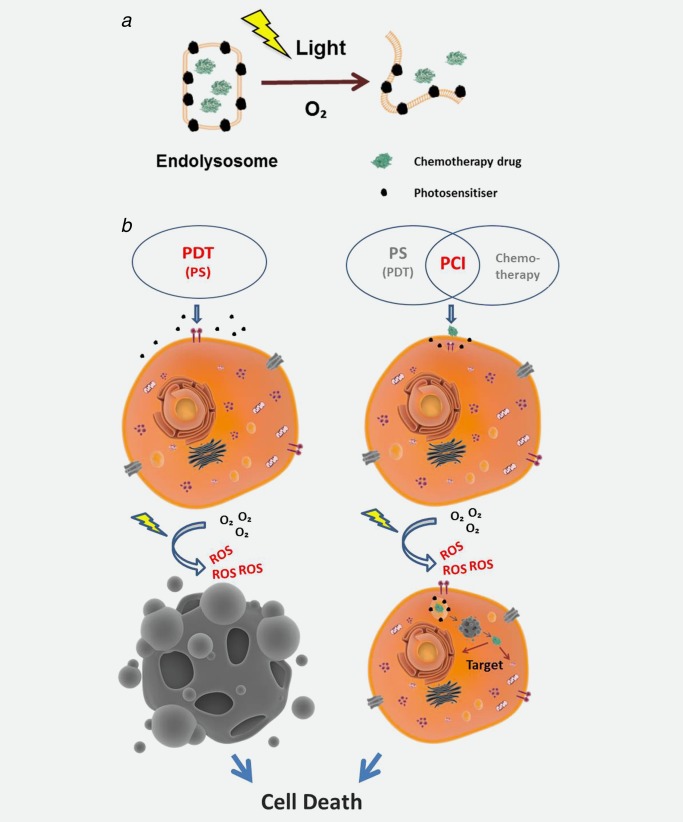
Photochemical Internalisation (PCI) *vs*. Photodynamic Therapy (PDT) The photooxidative damage in PCI is designed to be sub‐lethal but sufficient to release endolysomally entrapped agents such as cytotoxins so that they can reach their intended intracellular targets (*a*). Despite common features between PDT and PCI, these two treatments differ in key aspects (*b*). PCI is a site‐specific DDS^10^ therefore, unlike PDT, the photosensitisers employed in PCI are not used to cause direct cell death.^4^ [Color figure can be viewed in the online issue, which is available at wileyonlinelibrary.com.]

For *in vivo* studies, the PS and bioactive agent are administered systemically but the enhanced drug delivery induced by PCI is confined to the zone of illuminated tissue, and PCI is therefore a site‐specific technique. PCI has been successfully used for delivery of anti‐cancer cytotoxins such as bleomycin, which is now being tested clinically as discussed below. With improved intracellular delivery of the bioactive agents, the administered dose of the agent can be reduced which is important in the chemotherapy induced toxicity.

The development of PCI as a drug delivery technique stemmed in part from Photodynamic Therapy (PDT) which also involves the administration of a photosensitiser. Excitation of the PS results in the production of reactive oxygen species (ROS), particularly singlet oxygen which can oxidise important subcellular substrates including membrane components such as unsaturated lipids and cholesterol. Like PDT, PCI also relies on the presence of molecular oxygen for generation of ROS, although since oxygen solubility in organelle membranes is relatively high only strongly hypoxic cells should be unaffected by PCI.^13^ However these two treatments differ in key aspects (Fig. 1 *b*). PCI is a site‐specific drug delivery technique and thus, unlike PDT, the photosensitisers employed in PCI are not designed to induce a cytotoxic effect on their own. Besides, photooxidative damage to endolysosomes is not an efficient means of inducing cytotoxicity by itself.^14^ Because the PS is localised in endolysosomal membranes,^15^ photochemical damage to the drug cargo should be limited when light is applied after PS/drug administration, but if the drug is particularly sensitive to photochemical damage then a “light‐before” regime can be employed whereby the PS is administered and then light is applied before drug administration. Although this may appear to be counter‐intuitive, it is believed that the endolysosomal membranes which have already sustained photooxidative damage will fuse with the membranes of the endolysosomes containing the drugs thus rendering them more permeable.^16^

### Selection of photosensitisers for PCI

The photosensitiser should induce efficient generation of ROS and low aggregation propensity since monomeric PS is more photoactive, and exhibit strong absorption at red/near‐infrared wavelengths to enable deeper therapeutic efficacy. PCI is therefore distinct from other light‐triggered delivery systems which rely on UV excitation where tissue penetration is very limited. In practice, PDT is difficult to suppress in areas receiving the highest light dose, but PCI will dominate at the deeper regions of the tumour. Because the PS dose used for PCI is deliberately low, skin phototoxicity should be less likely.

There are several different photosensitisers currently available for PCI based on phthalocyanine, porphyrin or chlorin aromatic dye molecules, typically amphiphilic sulfonated derivatives where the sulfonate groups are substituted adjacently on one side of the aromatic macrocycle. The opposite unsubstituted side of the macrocycle is therefore relatively hydrophobic and can reside within the lipid bilayer, allowing the ionic substituted side to localise at the interface of the lipid/aqueous phase.

The resulting amphiphilic structure favours endolysosomal membrane localisation owing to uptake *via* adsorptive endocytosis with optimum localisation occurring after 18–24 hrs administration *in vitro* although longer times have been used *in vivo*. AlPcS_2a_ (aluminium phthalocyanine with two sulfonate groups) is a phthalocyanine‐based PS, was initially used for *in vivo* PCI owing to its strong absorption in the far‐red region (670 nm).^14^, ^17^ Recently, a disulfonated tetraphenylchlorin photosensitiser TPCS_2a_ (Amphinex®) has been developed, which contains far fewer regioisomers than AlPcS_2a_ and is therefore easier to prepare reproducibly.^18^ In 2011, it was proposed as an optimal PS for clinical PCI and is currently undergoing dose‐escalating phase I/II clinical trials in combination with bleomycin, as discussed below. TPCS_2a_ has suitable photophysical and photobiological properties for PCI^10^ and exhibits strong absorption at 650 nm. The porphyrin analogue TPPS_2a_ (meso‐tetraphenylporphin with two sulfonate groups on adjacent phenyl rings), has been extensively used *in vitro* with good results but is less suitable for clinical development since its red absorption is comparatively weak.^14^ Sulfonation is not the only method that has been used to confer amphiphilic properties. Wang *et al*. have shown that conjugation of a hydrophobic porphyrin to a cationic cell penetrating peptide rendered the porphyrin suitable for PCI.^19^

PCI is site selective owing to the light delivery, selective uptake of photosensitisers in tumours is generally insufficient to confer treatment selectivity. Enhanced selectivity may be conferred by passive targeting of the agents through the use of macromolecular drug carriers and the EPR effect (enhanced permeability and retention) or actively targeting the agents by conjugation with ligands such as antibodies. In the following section, we review cytotoxic cancer chemotherapy agents that have been used for PCI without active targeting followed by a review of results obtained using actively targeted drugs (see summary Table 1). The examples of drugs used fall into three categories: the glycopeptide antibiotics (bleomycin), anthracyclines (mitoxantrone, doxorubicin) and ribosome inactivating proteins (gelonin, saporin).

**Table 1:** Photochemical internalisation (PCI) experimental studies in different cancers

Cancer type	*In vivo/In vitro*	PS	Cytotoxic agents	References
Uterus cancer	*In vitro*	TPPS_2a_, ZnPc^1^, BPD‐MA^1^, 3‐THPP^2^, chlorin e_6_, AlPcS_2a_	Doxorubicin, (EGF‐) Saporin, (Cetuximab‐) Saporin, Gelonin	9, 20, 21, 22, 23, 24
Head and Neck cancer	*In vitro*	TAT‐TPP, TPPS_2a;_ AlPcS_2a_, mTHPC (and liposomal formulations)^1^	Saporin, (Polyamidoamine (PAMAM)) dendrimer‐Doxorubicin, (Polyamidoamine (PAMAM)) dendrimer‐ Saporin, Bleomycin	19, 25, 26, 27
Breast cancer	*In vitro*	TPCS_2a,_ AlPcS_2a,_ Hypericin^1^, TPPS_2a_	(Trastuzumab‐) Saporin, (EGF‐) Saporin, (IM7‐) Saporin, Mitoxantrone,(scFvMEL‐) rGelonin, (MOC31‐) Gelonin, MH3‐B1/rGel, Doxorubicin	21, 23, 28, 29, 30, 31, 32, 33, 34, 35
	*In vivo*	DPc	(DPc/m) Doxorubicin	36
Colon cancer	*In vitro*	TPCS_2a,_ AlPcS_2a,_ TPPS_2a,_ (5‐ALA)‐induced protoporphyrin IX (PpIX)^1^	(Trastuzumab‐) Saporin, (MOC31‐) Gelonin, (Cetuximab‐) Saporin, (IM7‐) Saporin	22, 28, 29, 34, 37
	*In vivo*	AlPcS_2a,_TPCS_2a,_ mTHPC^1^	Bleomycin, Gelonin, VEGF121/rGel	18, 38, 39, 40
Ovarian cancer	*In vitro*	TPPS_2a_	(Liposomally encapsulated) Saporin, (EGF‐) Saporin	21, 41
Sarcoma	*In vitro*	TPCS_2a,_ TPPS_2a_	(anti‐CD133‐) Saporin, (IM7‐) Saporin, Gelonin	34, 42, 43
	*In vivo*	TPCS_2a,_ AlPcS_2a_	Bleomycin, Gelonin	38, 44, 45, 46, 47
Bladder cancer	*In vitro*	Hypericin^1^, TPCS_2a,_ AlPcS_2a,_ TPPS_2a_	Mitoxantrone, Bleomycin, (scFvMEL‐) rGelonin	31, 35, 48
	*In vivo*	TPCS_2a_	No drug was used—Establishment of an optimal PCI treatment based on an orthotopic bladder cancer model	49
Glioma	*In vitro*	AlPcS_2a,_ TPPS_2a_	Bleomycin, (scFvMEL‐)rGelonin	35, 50
	*In vivo*	AlPcS_2a_	Bleomycin and ETXp (*Clostridium perfringens* epsilon protoxin)	51
Skin cancer	*In vitro*	TPPS_2a_, AlPcS_2a_, 3‐THPP^2^	(EGF‐) Saporin, (Cetuximab‐) Saporin, Gelonin, (MOC31‐) Gelonin	16, 21, 22, 29
	*In vivo*	AlPcS_2a_	(scFvMEL‐)rGelonin	35
Prostate cancer	*In vitro*	TPPS_2a_	(Cetuximab‐)Saporin, (IM7‐) Saporin	22, 34
Pancreatic cancer	*In vitro*	TPCS_2a_	(anti‐CD133‐)Saporin, (IM7‐) Saporin	34, 52
Lung cancer	*In vitro*	AlPcS_2a,_ 3‐THPP^2^	(MOC31‐)Gelonin	29

Several types of cancer have been subjected to PCI both *in vitro* and *in vivo* using different photosensitisers, and cytotoxic agents. In some cases the cytotoxin is actively targeted and the accompanying targeting ligand is shown in parentheses.

Although these photosensitisers will localise in organelle membranes, they are far less specific to endolysosomes and are thus less efficient as photosensitisers for Photochemical Internalisation.

In certain cases 3‐THPP was used as a negative control for PCI.

Abbreviations: TPCS_2a_—disulfonated tetraphenyl chlorin; TPPS_2a_—disulfonated tetraphenyl porphine; ZnPc—zinc phthalocyanine; BPD‐MA—benzoporphyrin derivative monoacid; 3‐THPP—tetra (3‐hydroxyphenyl) porphyrin; AlPcS_2a_—aluminium phthalocyanine disulfonate; DPc—dendrimer phthalocyanine; mTHPC—m‐tetra(hydroxyphenyl)chlorin.

## Cytotoxic Agents Used With PCI

To date, the majority of PCI studies published focus on macromolecular toxins, however, smaller cytotoxic chemotherapy drugs such as doxorubicin and bleomycin, have also been studied. Bleomycin is currently being used in clinical trials of PCI. In this section we review results using these agents together with studies on macromolecular carriers incorporating cytotoxins.

### Glycopeptide antibiotics and anthracyclines

Bleomycin is a hydrophilic glycopeptide antibiotic with a relatively high molecular weight (1.4 kDa) which favours uptake *via* endocytosis. Cytotoxicity is caused by single and double‐stranded DNA damage. Given alone, bleomycin can result in pneumonitis and subsequent lung fibrosis at high doses.^38^ Enhanced intracellular delivery using PCI is therefore attractive as a way to reduce the bleomycin dose required, and potentially the number of treatment cycles.

Berg *et al*. in 2005 performed PCI both in *in vitro* and *in vivo* human sarcoma, human colorectal adenocarcinoma and murine colon carcinoma models. TPPS_2a_ (0.7 µg mL^−1^) and bleomycin (0.14 IU mL^−1^) significantly enhanced cytotoxicity by a factor of three compared to bleomycin alone *in vitro* 38 using illumination after bleomycin administration. The combination of PCI with bleomycin and AlPcS_2a_, resulted in significant tumour growth delay compared to control groups without PCI. Furthermore, the initial weight loss seen in bleomycin treated animals, was reversed in the PCI group.^38^ In a key *in vivo* study, Norum et al.^45^ demonstrated in a murine fibrosarcoma model that the tumour periphery was more susceptible to damage using bleomycin PCI compared to PDT alone with AlPcS_2a_. Using histology, a smaller area of viable peripheral tissue was seen post‐PCI for the treated animals than for PDT alone. The greater cell killing in this highly proliferating area was not due to higher levels of AlPcS_2a_ as confirmed in fluorescence studies.

PCI using bleomycin has also been investigated for bladder and brain cancer. Arentsen *et al*. demonstrated the advantage of bleomycin PCI using a range of chemotherapeutic agents in several human and non‐human bladder cancer cell lines *in vitro*.^48^ Enhanced cell kill using bleomycin PCI was also observed in glioma cell monolayers and multicellular spheroids.^50^ *In vivo* studies showed improved survival of animals bearing the F98 glioma model using a combination of bleomycin PCI and an epsilon prototoxin which is known to disrupt the blood‐brain barrier *versus* controls.^51^ The combination of bleomycin with external‐beam radiotherapy has also been studied, which elicited a greater delay in tumour progression, and could enable a reduction in the required dose of ionising radiation.^46^ This may be relevant to future clinical studies where a combination of therapeutic techniques is employed.

As discussed above, amphiphilic photosensitisers are generally used for PCI but in a recent *in vitro* study on human head and neck cancer cell lines, bleomycin PCI has been shown to be effective when a lipophilic photosensitiser is administered using liposomal formulations which should favour uptake by endocytosis. However for this approach to work effectively *in vivo* the photosensitiser would have to remain confined to the liposome once in circulation.^27^

PCI has also been used successfully with anthracycline drugs, doxorubicin (0.5 kDa) and mitoxantrone (0.4 kDa), which inhibit DNA and RNA synthesis. Although these are relatively small molecules, they are weak bases which may be retained within acidic lysosomes due to ion‐trapping of the protonated form. In 2006, Lou *et al*. compared PCI in doxorubicin sensitive and doxorubicin resistant MCF‐7 breast cancer cell lines and showed that PCI overcame endosomal entrapment of the doxorubicin in the resistant cell line by promoting transport of doxorubicin to cell nuclei.^30^ PCI was also effective in mitoxantrone‐resistant cells using hypericin.^31^ Multidrug‐resistance in uterine sarcoma cells was shown to be abrogated using PCI by Selbo *et al*.,^24^ and the same team later hypothesised that the endolysosomal localisation of the PS could protect them from efflux *via* the ATP‐binding cassette transporter involved in MDR (ABCG2).^32^

### Macromolecular toxins—Ribosome inactivating proteins (RIP)

Type 1 ribosome inactivating protein (RIP) inhibitors such as gelonin and saporin (approximately 30 kDa MWt), which are plant‐derived are candidate chemotherapy agents but require cytosolic delivery. Gelonin and saporin are both highly toxic but subject to endolysosomal sequestration and degradation, and have therefore been widely investigated for PCI studies.^9^, ^16^, ^20^, ^34^, ^42^, ^44^ Berg *et al*. used the NHIK 3025 cervical carcinoma cell line exposed to a combination of gelonin and photosensitisers (TPPS_2a_ or AlPcS_2a_).^9^ Up to a 200‐fold increase in cell death was seen compared to either treatment alone, thus confirming a synergistic effect. A similar study was carried out by Selbo *et al*.,^20^ in which gelonin was released from endosomes after PCI; yet, if PCI was delayed and gelonin was found in lysosomes rather than early endosomes, cell killing efficiency was reduced owing to drug degradation within lysosomes.^20^ THX melanoma cells were also exposed to gelonin and AlPcS_2a_ to determine the difference in gelonin‐induced cytotoxicity using a light—“before” or —“after” approach. A light‐before strategy significantly enhanced gelonin's cytotoxicity^16^ in agreement with the aforementioned findings.^20^ Nevertheless, the use of either PCI strategy will depend on specific conditions of the study, and the compounds being delivered.^16^, ^21^, ^22^, ^28^ Dietze *et al*. completely eradicated sarcoma induced in mice in 50% of gelonin PCI treated animals, and showed significantly delayed tumour regrowth in the remaining 50% compared to PDT or non‐treated groups.^44^ Saporin is another type 1 RIP inhibitor which has been studied with enhanced cytotoxicity observed using PCI.^18^, ^19^ Additional studies on saporin in relation to targeted PCI will be reviewed below.

### Macromolecular drug carriers

PCI is well suited for the delivery of macromolecular drug carriers, such as dendrimers and other nanocarriers, which owing to their size are taken up by endocytosis, thereby limiting the efficacy of the drug which then has to escape from the endolysosomes to reach its target site. Fretz *et al*. investigated cytotoxicity induced using liposomes containing toxins such as saporin by PCI.^41^ Cationic liposomes exhibited the highest cellular uptake and resulted in the greatest reduction in cell viability, whereas no cytotoxicity was observed when saporin was delivered without PCI using liposomes or on its own.^41^ Similarly, a better outcome was seen when loading doxorubicin in PEG liposomes also containing chlorin e6 incorporated within the membrane, as opposed to liposomes loaded with doxorubicin or chlorin e6 on their own.^53^

Pasparakis *et al*. recently developed polymeric nanocarriers based on ketals which were co‐loaded with camptothecin (0.35 kDa) and the photosensitiser haematoporphyrin which were administered to HeLa cells.^54^ The structure was designed to be degradable in acidic lysosomes thereby releasing the lipid soluble haematoporphyrin to localise in membranes for PCI. A synergistic enhancement in cytotoxicity was observed using PCI.^54^ A similar approach has also been applied for gene transfection using biodegradable polyamino acid carriers.^55^ A nanocarrier based on chitosan bearing a covalently bound porphyrin resulted in successful PCI transfection of EGFP in human colon carcinoma cells *in vitro*.^56^ The same method could be applied for cytotoxic drug delivery. The authors hypothesised that in the presence of lipid membranes, the carrier structure was able to unfold allowing the hydrophobic porphyrin to be inserted into the lipid membrane. PCI could similarly be applied to the delivery of small molecule carrier systems (SMoC) in the delivery of siRNA or miRNA.^57^

## Targeted Strategies

### Endothelial growth factor receptor (EGFR)

EGFR targeted PCI has been the focus of several studies, where the RIP inhibitor saporin has been conjugated to anti‐EGFR molecules.^21^, ^22^, ^32^ EGFR up‐regulation on the cell surface has also been associated with drug resistance.^58^, ^59^ Weyergang *et al*. in 2006^21^ and Selbo *et al*. in 2012^32^ showed synergistic cytotoxicity using PCI delivery of saporin when targeted with EGF against EGFR positive breast, ovarian and skin carcinoma cells.^21^, ^32^ Similarly, Cetuximab®‐saporin PCI has also been used, directed against EGFR in colorectal and prostate cancer cells, where it was shown that targeted‐saporin led to improved cytotoxicity as opposed to almost no effect when exposing cells to the same concentration range of untargeted streptavidin‐saporin.^22^

### Endothelial glycoprotein‐2

Endothelial glycoprotein‐2 (EGP‐2), is overexpressed in most carcinomas.^29^ Gelonin has been covalently linked to MOC31 (an antibody recognising EGP‐2) and has been used in several cancer models.^29^, ^37^, ^39^ A PCI‐based synergy was found between gelonin and two photosensitisers (TPPS_2a_ and AlPcS_2a_), with a greater effect seen with the targeted MOC31‐gelonin in a small cell lung carcinoma cell line (NCI‐H146). Lower cytotoxicity was seen with MOC31‐gelonin alone, but were equally efficient on EGP‐2 antigen negative cells.^29^

The same study was performed using WiDr human colorectal cells *in vitro* 37 using 5‐aminolaevulinic acid‐induced porphyrin photosensitisation. MOC31‐gelonin was combined with AlPcS_2a_ to treat mice bearing subcutaneous WiDr tumours, where 20 days after PCI treatment in 6/9 cases tumours were completely eliminated. Moreover, no weight variation was seen in the animals and skin damage was resolved 3 weeks after therapy.^39^

### Human epidermal growth factor receptor 2

HER2 is overexpressed in ∼25% of all breast cancer cases and it is used to determine progression and prognosis.^60^, ^61^ Based on this, Berstad *et al*. in 2012^28^ combined trastuzumab (Herceptin®)‐saporin and a chlorin photosensitiser to compare PDT *versus* PCI induced cell killing on either HER2^+^ or HER2^−^ breast cancer cells.^28^ Light administration post exposure of cells to (Herceptin®)‐saporin in PCI was far superior in cell killing compared to PDT and the PCI light‐before treatment. The authors hypothesised that photooxidative damage to HER2 with the light‐before treatment resulted in less efficient drug delivery.^28^ Gelonin was similarly targeted against several breast cancer cell lines showing different levels of expression of HER‐2 receptor,^33^ and it was shown that in addition to toxin uptake, relative cellular sensitivity to the toxin is also an important factor affecting PCI efficacy.^33^

### Progenitor marker gp240

The progenitor marker gp240 has been found to be relevant in lobular breast carcinomas, gliomas and melanomas.^62^ Accordingly, Selbo *et al*.^35^ used a fusion toxin consisting of a recombinant single chain antibody against gp240 and gelonin (scFvMEL/rGel), which was delivered by PCI using either of two photosensitisers (AlPcS_2a_ and TPPS_2a_). This fusion toxin was used to demonstrate the potential of PCI for skin cancer treatment *in vivo* (A‐375 cells), as well as malignant glioma (U87MG) and lobular breast carcinoma (MA11) cells *in vitro*.^35^ Furthermore, the synergistic PCI effect of scFvMEL/rGel was largely more effective than PDT‐, chemo‐ or non‐ treated groups.^35^ In the T24 bladder cell line (gp240 negative) no difference was observed between targeted and non‐targeted PCI. Equally, as already suggested by Berstad *et al.,* 28 cell antigens can be damaged by light exposure, resulting in a lower uptake of antibody‐fused toxins; therefore this factor needs to be considered when choosing an appropriate PCI protocol.

### Cd133

PCI has been used to successfully target cells expressing CD133, a surface molecule which has been described as a marker for stem‐like cancer cells exhibiting resistance to chemotherapeutic agents and resulting in a poor prognosis.^43^, ^52^ Fibrosarcoma and liposarcoma cells *in vitro* were exposed to saporin bound to antibodies against two different epitopes of CD133, combined with TPCS_2a_ and light.^43^ Administration of anti‐CD133 caused some cytotoxicity on its own, which could be related to iron uptake pathways and induction of dedifferentiation in cells. PCI‐treated cells *in vitro*, were able *to* delay *in vivo* tumour growth in 2/3 cases and no tumour regrowth occurred in 1/6 cases. PDT studies *in vitro* seemed to trigger cellular proliferative signals, which would correlate with the tumour regrowth observed *in vivo*.^43^

A similar study was conducted on WiDr colorectal cells (CD133^high^, 95% expression), a pancreatic cancer cell line with a small population of CD133^+^ (BxPC‐3, <4% expression) and a prostate cancer cell line which is CD133^low^ expressing, (Du‐145, 0.7% expression).^52^ In the colorectal cancer cells, CD133‐saporin PCI was able to almost completely eliminate viability at a dose as low as 10 fM; yet, results with cells exposed to 0.8 pm of unconjugated saporin, were similar to PDT.^52^ Pancreatic cancer cells (BxPC‐3), showed a 50% reduction in cell viability, which seemed to be exclusively caused by the bound anti‐CD133. After PCI, cell viability was further reduced by 90%, despite showing resistance to PDT. Once again these results correlate with the exposure of DU 145 cells (lacking CD133) to anti‐CD133‐saporin, in which case, toxicity induced by either toxin alone or targeted was similar.^52^ Importantly, high proliferative capacity and tumour formation ability was confirmed with cells that highly express CD133 *in vitro* and *in vivo*; thus, targeted PCI could be in this case be a highly effective treatment against aggressive, and rapidly metastasising tumours.

Additional cancer stem cell markers such as CD44 or epithelial cell adhesion molecule (EpCAM) have also been the target of PCI treatment, confirming the efficacy of relevant immunotoxins based on saporin.^34^, ^63^

## Potential Advantages of PCI

### Overcoming chemotherapy‐drug resistance

In previous sections we have reviewed several studies showing that PCI is effective in treating drug‐resistant cell lines.^23^, ^24^, ^28^, ^30^, ^31^, ^32^, ^34^, ^36^ It has also been proposed that photooxidative damage to efflux pumps can counteract chemotherapy resistance. An example of this was described by Lu *et al*. who used encapsulation of PS in micellar structures, and in addition to overcoming doxorubicin resistance in MCF‐7 resistant cells both *in vitro* and *in vivo*, they found in the doxorubicin resistant tumours, the greatest antitumour effect was seen by treating with light‐before PCI. It was thought that P‐glycoprotein membrane transporter shutdown may facilitate intracellular and nuclear accumulation of doxorubicin.^36^

### Potential as an adjuvant to surgical resection

Within a tumour non‐proliferating cells are usually found in the centre, whereas proliferating cells generally reside in the periphery of an actively growing tumour mass. Norum *et al*. demonstrated higher efficacy of bleomycin PCI compared to PDT towards the periphery of a murine fibrosarcoma.^45^ In a subsequent study using PDT or PCI to treat the tumour bed after surgical resection, Norum *et al*. demonstrated that PCI with surgery was able to induce a significant delay in tumour growth compared to surgery, and PDT in combination with surgery.^47^ The lower efficacy of PDT with surgery is consistent with the lower sensitivity of cells at the tumour margin to PDT compared to PDT. This study suggests that PCI may have a role as an adjuvant to surgical resection to ensure complete tumour removal.^47^

### Antivascular effects of PCI

For cancer therapy PCI has generally been aimed at enhancing delivery within the tumour cells. However, recent work has shown that PCI is also capable of affecting vascular structures^40^, ^45^, ^64^ which is relevant to treatment of tumours containing drug resistant cells which will die if the tumour microvasculature is damaged. Vascular shutdown has been widely described as an important component of the PDT effect. In 2009, Norum *et al*. also confirmed vascular shutdown post‐PCI occurring at a later stage than for PDT.^45^ Accordingly, endothelial cells as potential PCI targets have been studied. The combined effect of either photosensitiser TPPS_2a_ or AlPcS_2a_ with saporin was recently compared using human vascular endothelial cells (HUVECs) and fibrosarcoma cells.^64^ Both photosensitisers were more efficiently taken up by HUVEC cells and found to be located in endocytic vesicles. These results suggest more attention should be given to vascular endothelial cells during PCI treatment given the capacity of both PDT and PCI to act on endothelial cells, which in a physiological environment could be translated into vascular shutdown.

## Clinical Work With PCI

A single‐centre (University College Hospitals—UCH) dose‐escalation phase I clinical trial was successfully completed on 22 patients in 2013 with superficial skin and head and neck neoplasms combining bleomycin and Amphinex® (ClinicalTrials.gov identifier: NCT00993512). No adverse reactions were found so a multi‐centre phase II study (ClinicalTrials.gov identifier: NCT01872923) is now being carried out between several European centres (UK, Netherlands, France, Germany and Lithuania), which will focus on both superficial and deeper head and neck tumours using interstitial illumination. In addition, a phase I/II dose escalation study of PCI of gemcitabine using Amphinex®, followed by gemcitabine/cisplatin chemotherapy has recently commenced for advanced inoperable cholangiocarcinomas (ClinicalTrials.gov identifier: NCT01900158).

## Conclusions and Future of PCI

Many research groups worldwide are investigating PCI, and there is growing evidence supporting its potential as a drug delivery system in cancer treatment. PCI is a very versatile technique which has been shown to be effective for delivery of drugs exhibiting diverse physicochemical properties and sizes and may find application for cytosolic delivery in the emerging field of “nanomedicine.” Site‐specific light application to the target lesion results in the focal delivery of therapeutic compounds, thereby minimising damage to normal adjacent tissue^8^, ^15^ although this means that PCI is only suitable for local treatment of cancer. PCI could potentially widen the range of chemotherapy options to include relatively cytotoxic chemotherapy drugs whose dosages could be lowered while maintaining their therapeutic effect. Further work is required on optimising light dosimetry for PCI since it relies on adequate light doses reaching the tumour extremities, and on optimisation of the drug and photosensitiser doses and timing of administration. These complications will have to be addressed in clinical trials in order for PCI to gain widespread acceptance. Photooxidative damage induced by PDT has been shown to elicit an immune response, albeit variable in nature,^65^, ^66^, ^67^, ^68^, ^69^, ^70^ but it will be important to establish whether PCI is also able to trigger such a response which could help improve tumour control. In conclusion, PCI is a promising means of enhancing chemotherapy and like PDT may find application for focal treatment of a wide range of solid tumours including pancreatic and prostate cancer.
